# Enhancing Exposure of HIV-1 Neutralization Epitopes through Mutations in gp41

**DOI:** 10.1371/journal.pmed.0050009

**Published:** 2008-01-03

**Authors:** Catherine A Blish, Minh-An Nguyen, Julie Overbaugh

**Affiliations:** 1 Division of Human Biology, Fred Hutchinson Cancer Research Center, Seattle, Washington, United States of America; 2 Division of Infectious Diseases, University of Washington, Seattle, Washington, United States of America; New York University School of Medicine, United States of America

## Abstract

**Background:**

The generation of broadly neutralizing antibodies is a priority in the design of vaccines against HIV-1. Unfortunately, most antibodies to HIV-1 are narrow in their specificity, and a basic understanding of how to develop antibodies with broad neutralizing activity is needed. Designing methods to target antibodies to conserved HIV-1 epitopes may allow for the generation of broadly neutralizing antibodies and aid the global fight against AIDS by providing new approaches to block HIV-1 infection. Using a naturally occurring HIV-1 Envelope (Env) variant as a template, we sought to identify features of Env that would enhance exposure of conserved HIV-1 epitopes.

**Methods and Findings:**

Within a cohort study of high-risk women in Mombasa, Kenya, we previously identified a subtype A HIV-1 Env variant in one participant that was unusually sensitive to neutralization. Using site-directed mutagenesis, the unusual neutralization sensitivity of this variant was mapped to two amino acid mutations within conserved sites in the transmembrane subunit (gp41) of the HIV-1 Env protein. These two mutations, when introduced into a neutralization-resistant variant from the same participant, resulted in 3- to >360-fold enhanced neutralization by monoclonal antibodies specific for conserved regions of both gp41 and the Env surface subunit, gp120, >780-fold enhanced neutralization by soluble CD4, and >35-fold enhanced neutralization by the antibodies found within a pool of plasmas from unrelated individuals. Enhanced neutralization sensitivity was not explained by differences in Env infectivity, Env concentration, Env shedding, or apparent differences in fusion kinetics. Furthermore, introduction of these mutations into unrelated viral Env sequences, including those from both another subtype A variant and a subtype B variant, resulted in enhanced neutralization susceptibility to gp41- and gp120-specific antibodies, and to plasma antibodies. This enhanced neutralization sensitivity exceeded 1,000-fold in several cases.

**Conclusions:**

Two amino acid mutations within gp41 were identified that expose multiple discontinuous neutralization epitopes on diverse HIV-1 Env proteins. These exposed epitopes were shielded on the unmodified viral Env proteins, and several of the exposed epitopes encompass desired target regions for protective antibodies. Env proteins containing these modifications could act as a scaffold for presentation of such conserved domains, and may aid in developing methods to target antibodies to such regions.

## Abstract 2

Julie Overbaugh and colleagues analyze an HIV strain with high susceptibility to antibody neutralization and identify two gp41 envelope mutations that confer this sensitivity by exposing multiple neutralization epitopes.

## Editors' Summary

**Background.:**

In 1984 when scientists identified human immunodeficiency virus (HIV)—the cause of acquired immunodeficiency syndrome (AIDS)—many experts believed that a vaccine against HIV infection would soon be developed. Nearly 25 years later, there is still no such vaccine and with about 2.5 million new HIV infections in 2007, an effective vaccine is urgently needed to contain the AIDS epidemic. Vaccines provide protection against infectious diseases by priming the immune system to deal quickly and effectively with viruses and other pathogens. Vaccines do this by exposing the immune system to an immunogen—a fragment or harmless version of the pathogen. The immune system mounts a response against the immunogen and also “learns” from this experience so that if it is ever challenged with a virulent version of the same pathogen, it can quickly contain the threat. Many vaccines work by stimulating an antibody response. Antibodies are proteins made by the immune system that bind to molecules called antigens on the surface of pathogens. Antibodies that inactivate the invader upon binding to it are called “neutralizing” antibodies.

**Why Was This Study Done?:**

Several characteristics of HIV have hampered the development of an effective vaccine. An “envelope” protein consisting of two subunits called gp120 and gp41 covers the outside of HIV. Many regions of this protein change rapidly, so the antibody response stimulated by a vaccine containing the envelope protein of one HIV variant provides little protection against other variants. However, other regions of the protein rarely change, so a vaccine that stimulates the production of antibodies to these “conserved” regions is likely to provide protection against many HIV variants. That is, it will stimulate the production of broadly neutralizing antibodies. Unfortunately, it has been difficult to find HIV vaccines that do this, because these conserved regions are often hidden from the immune system by other parts of the envelope protein. In this study, the researchers investigate the envelope protein of an HIV-1 variant they have isolated that is highly susceptible to inactivation by antibodies specific for these conserved regions. Comparing the envelope protein of this sensitive virus to closely related envelope proteins that are resistant to neutralization could identify features that might, if included in an envelope protein immunogen, produce a vaccine capable of generating broadly neutralizing antibodies.

**What Did the Researchers Do and Find?:**

The researchers isolated a subtype A HIV-1 variant from a newly infected woman in Kenya that was efficiently neutralized by monoclonal antibodies (antibodies made by cells that have been cloned in the laboratory). These antibodies were specific for several different conserved regions of gp41 and gp120. The isolate was also neutralized by antibodies in blood from HIV-1-infected people. The envelope protein of the sensitive variant was the same as that of a resistant variant isolated at the same time from the woman, except for four amino acid changes in conserved regions of gp41 (proteins are made from long strings of amino acids). Using a technique called site-directed mutagenesis, the researchers introduced these amino acid changes into envelope proteins made in the laboratory and determined that just two of these changes were responsible for the neutralization sensitivity of the HIV-1 variant. The introduction of these two changes into the neutralization resistant variant and into the unrelated envelope sequences of another subtype A (common in Africa) HIV-1 variant and a subtype B HIV-1 (common in Europe and the Western Hemisphere) variant increased the sensitivity of all these viruses to antibody neutralization.

**What Do These Findings Mean?:**

These findings show that two amino acid changes in gp41 of a neutralization-sensitive HIV-1 variant are responsible for the sensitivity of this variant to several neutralizing antibodies. The finding that the inclusion of these changes in the envelope protein of neutralization-resistant HIV-1 variants greatly increases their sensitivity to neutralizing antibodies indicates that the normally shielded regions of the protein are somehow made accessible to antibody by these changes. One possibility is that the amino acid changes might modify the overall shape of the envelope protein, thus exposing multiple, normally hidden regions in the HIV-1 envelope protein to antibodies. Importantly, these findings open up the possibility that the inclusion of these modifications in envelope-based immunogens might improve the ability of vaccines to generate broadly neutralizing antibodies against HIV-1.

**Additional Information.:**

Please access these Web sites via the online version of this summary at http://dx.doi.org/10.1371/journal.pmed.0050009.

- Information is available from the US National Institute of Allergy and Infectious Diseases on HIV infection and AIDS
- HIVInSite has comprehensive information on all aspects of HIV/AIDS, including links to resources dealing with HIV vaccine development
- Information is available from Avert, an international AIDS charity, on all aspects of HIV and AIDS, including HIV vaccines
- The US Centers for Disease Control and prevention provides information on HIV/AIDS including information on its HIV vaccine unit (in English and some information in Spanish)
- The AIDS Vaccine Clearinghouse provides clear information about HIV vaccine science, research and product development
- The International AIDS Vaccine Initiative also provides straightforward information about the development of HIV vaccines

## Introduction

The generation of broadly neutralizing antibodies by a vaccine is a high priority in the global fight against HIV-1. Proof-of-concept experiments in primate models have demonstrated that passively acquired HIV-1–specific antibodies capable of neutralizing the infecting strain can prevent infection through mucosal and intravenous routes [1–5]. However, the generation of antibodies that are able to neutralize transmitted strains of HIV-1 has proved extremely challenging; such antibodies are uncommon in naturally infected individuals and methods to elicit them by vaccination have not been fruitful [6]. Developing a method to direct antibody responses to conserved regions of the HIV-1 Envelope (Env) protein may allow antibodies to better neutralize the vast array of diverse HIV-1 variants currently circulating.

Unfortunately, HIV-1 has evolved a number of mechanisms with which it hampers the generation of neutralizing antibody responses against conserved regions of the Env protein. This Env protein, gp160, is cleaved into two subunits, gp120 (the surface subunit) and gp41 (the transmembrane subunit), that trimerize and are noncovalently associated on the surface of the virion. The gp120/gp41 complex evades antibody responses by shielding conserved regions with both variable loops and with the addition of potential N-linked glycosylation sites [7–9]. Antibody access to conserved regions is further limited because viral entry is a stepwise process involving conformational changes that lead to only transient exposure of conserved domains such as the coreceptor binding site [7–9]. The few broadly neutralizing antibodies that have been discovered have unusual structural features, providing somewhat limited insight into how to overcome these evasion mechanisms. For instance, the monoclonal antibody (MAb) b12 has an extended CDR3 loop with which it reaches far into the CD4 receptor binding site of HIV-1 Env [10,11]. The MAbs 2F5 and 4E10, which target conserved epitopes within the membrane-proximal external region (MPER) of gp41, are unusually hydrophobic and may even be autoreactive [12,13]. The unusual structural features of these MAbs likely reflect the difficulty that antibodies face in gaining access to conserved regions of HIV-1.

Newly transmitted strains of HIV-1 may provide unique insights into how to expose these conserved epitopes to the immune system. Because antibody responses to HIV-1 take several months to mature, these early variants exist in the absence of a neutralizing antibody response, and may therefore contain structural features that are good targets for broad neutralization, but are normally selected against later in infection. Some, though not all, early strains of HIV-1 appear to be highly susceptible to neutralization [14–19], suggesting that at least a subset have some exposed neutralization epitopes. Using samples collected as part of a longitudinal cohort study in which participants are identified near the time of their infection, we identified an individual who had early HIV-1 variants with dramatically different neutralization sensitivities [17]. We therefore compared an early subtype A HIV-1 variant that was unusually susceptible to neutralization to a variant from the same individual that was highly neutralization resistant, in order to identify features of the viral Env protein that led to the exposure of conserved neutralization domains.

## Materials and Methods

### Cloning and Generation of Wild-Type and Mutant HIV-1 Viral Envelope Proteins

The full-length subtype A HIV-1 viral *Envs* Q461d1 and Q461e2 were cloned directly from peripheral blood mononuclear cells (PBMCs) 28 d postinfection from one high-risk study participant in Mombasa, Kenya, and have been described [17,20]. The Q769b9 viral *Env* was cloned 56 d postinfection from PBMCs from another participant within the same study [17,20]. The subtype B HIV-1 *Env* from YU-2 was cloned as described [20] from the *YU-2* gene donated to the NIH AIDS Research and Reference Reagent program by Drs. Hahn and Shaw. To introduce amino acid changes into the neutralization-resistant backgrounds, we used site-directed mutagenesis according to the instructions for Quik-Change site-directed mutagenesis kit (Stratagene). Briefly, using a plasmid encoding a neutralization-resistant HIV-1 Env protein as a template, we designed primers containing the desired mutation. The primers used and PCR conditions are described in Table S1. We amplified the plasmid template with each forward and reverse primer using Pfu Turbo (Stratagene), digested the reaction with DpnI (New England Biolabs), and transformed into DH5α cells by electroporation. Colonies were screened for presence of HIV-1 Env by restriction digestion with MluI and NotI (New England Biolabs), and the entire HIV-1 Env variant was sequenced using BigDye (Applied Biosystems). In order to generate double mutants, plasmid containing a single mutation was used as template.

### Determination of the Frequency of Amino Acid Changes at Positions 569 and 675

In participant Q461 at 28 d postinfection, 18 full-length *env* clones (including Q461d1 and Q461e2) were previously amplified by limiting dilution as described [20]. These sequences were aligned using Sequencher (Gene Codes Corporation) and the predicted amino acid sequence was determined in order to evaluate the frequency of the mutant allele. To evaluate the frequency of the mutant alleles at a later time point, DNA was extracted from ∼5 million viable frozen PBMCs taken from the same individual at 3.7 y postinfection using the QIAamp DNA Blood Mini kit (Qiagen). For each sample, the HIV-1 proviral copy number was estimated using real-time quantitative PCR that amplified a 165 bp region of *pol* [21,22]. From an estimated 1 copy of proviral template, separate nested PCRs were used to amplify a 417 bp region of gp41 flanking the two mutations. PCR primers for round 1 were: env1613-27F: 5′-CGGTACAGGCCAGAC-3′ and reverse env2120-36R 5′-GTATCCCTGCCTAACTC-3′. Round 2 primers were env1665-81F 5′-GCTGAAGGCTATAGAGG-3′ and env2065-82R 3′-TCCTATTAAGCCTCCTAC-5′. PCR conditions for both rounds were: 94 °C for 4 min; followed by 35 cycles of 94 °C for 1 min, 55 °C for 30 s, 72 °C for 1 min; then 72 °C for 8 min; and a 4 °C hold. The PCR products were purified using Exo-SAP (Amersham Biosciences), directly sequenced using BigDye, and analyzed for the presence of the mutant alleles.

### Plasma Samples and Antibodies

The plasma sample from participant Q461 was taken at 3.7 y postinfection and was collected as part of the longitudinal study of HIV-1 among high-risk women in Mombasa, Kenya [23,24]. The plasma pool was generated by pooling plasma collected between 1998 and 2000 from 30 HIV-1-positive individuals in Mombasa, Kenya [17]. Most of these individuals were infected with subtype A HIV-1, and a small subset were infected with subtypes D and C [25]. The MAb IgG1 b12 (b12) was provided by Dennis Burton (The Scripps Research Institute, La Jolla, California, United States), the MAbs 2G12, 2F5, and 4E10 were provided by Hermann Katinger (Polymun Scientific, Vienna, Austria), and soluble CD4 (sCD4) was from Invitrogen.

The study was approved by the ethical review committees of the University of Nairobi, the University of Washington, and the Fred Hutchinson Cancer Research Center, and all participants gave verbal informed consent.

### Generation of Pseudoviruses and Infectivity Assays

Pseudoviruses were generated by cotransfection of 293T cells with the viral Env protein and with Q23Δenv, a subtype A HIV-1 proviral clone with a partial deletion in Env, as described [20]. After 48 h, viral supernatants were harvested and sterile-filtered through a 0.2 μm filter to remove cellular debris, aliquotted, and frozen. Infectious titers were determined by infecting TZM-bl cells (NIH AIDS Research and Reference Reagent Program) for 48 h in the presence of 20 μg/ml DEAE-dextran, and counting blue foci after staining fixed cells for β-galactosidase activity [26]. As a control, experiments were also performed in the absence of 20 μg/ml DEAE-dextran. Quantification of p24 levels in viral supernatants was performed using a HIV-1 p24 Antigen ELISA Kit (ZeptoMetrix Corporation) according to manufacturer's instructions. Two pseudoviruses, Q769b9.TAIV and YU-2.TA, were of low infectious titer, and were concentrated using Amicon Ultra 100K Filter Devices (Millipore) prior to aliquotting and freezing. Purification of virus away from shed Env was performed by centrifuging at 67,000*g* through a cushion of 20% sucrose in PBS for 90 min at 4 °C [17]. Infectious titer was determined before and after purification, and approximately 50% of the loaded infectious particles were recovered.

All neutralization assays were performed in triplicate with at least two independent preparations of pseudoviral stock, as described [17,27]. Briefly, 500 infectious particles of pseudovirus were mixed with serial dilutions of MAb or plasma for 1 h, then TZM-bl indicator cells were added and infection levels were determined by assessing β-galactosidase activity after 48 h. Median inhibitory concentrations (IC50s) were defined as the concentration of MAb or sCD4, or the reciprocal dilution of plasma that resulted in 50% inhibition, calculated as described [17,27]. To determine the fold-difference in neutralization to MAbs and sCD4, the IC50 value of the sensitive mutant was divided into the IC50 value for the resistant variant. When the virus was not neutralized at 50% even at the highest neutralizing antibody concentration tested, the highest concentration tested (25 μg/ml) was used, with the resulting calculation then revealing the smallest possible difference. For plasma, the fold-difference was calculated by dividing the IC50 value of the resistant variant into that of the mutant. The lowest dilution of plasma (12.5) was used as the IC50 value when neutralization of <50% was observed. Inhibition by enfuviritide (T-20, NIH AIDS Research and Reference Reagent Program) was performed exactly as for neutralization assays, except that dilutions of enfuviritide were used instead of plasma or MAb.

### Pseudoviral Envelope Western Blotting and Quantification

To compare Env content between pseudoviral preparations, purified pseudoviral preparations of equal titer were subjected to Western blotting as described [17]. Briefly, pseudoviral stocks were concentrated 8-fold with Amicon Ultra 100K Filter devices, then an aliquot was removed for titration. The remaining pseudoviral stock was pelleted by microcentrifugation at 16,000*g* for 90 min at 4 °C, and the viral pellet was resuspended in SDS loading buffer with reducing agent (Invitrogen), boiled for 3 min, and stored at −20 °C. When titration results were available, an equivalent number of infectious particles from each pseudoviral preparation was resolved on a 4%–12% gradient Bis-Tris polyacrylamide gel, followed by electrotransfer to a nitrocellulose membrane. Western blotting and protein quantification was performed using the Odyssey infrared imaging system (LI-COR Biosciences). A rabbit polyclonal antisera to HIV-1 was used as a primary antibody [28], and 700DX-conjugated goat-anti-rabbit IgG was used as a secondary antibody (Rockland Immunochemicals). Identification of gp160 was confirmed by double staining with the MAb 2F5 as a primary and 800DX-conjugated goat-anti-human IgG (Rockland Immunochemicals) as a secondary antibody.

### Statistical Analyses

Statistical analyses were performed using Intercooled Stata 8.0. In order to evaluate whether Env protein concentration correlated with neutralization sensitivity, we performed Spearman rank correlations comparing the relative Env protein concentration of each of the five Q461 variants with the IC50 values of these variants to each monoclonal antibody and plasma sample. These comparisons were performed using the mean values for each strain from multiple experiments, in order to prevent each strain from entering the analysis multiple times. For instance, we first correlated the gp120 concentration of Q461d1, Q461e2, Q461e2.TA, Q461e2.IV, and Q461e2.TAIV with the 2F5 IC50 by Spearman rank correlation. We then performed the same correlation using the 4E10 IC50, and so forth for b12, sCD4, autologous plasma, and heterologous plasma. The same analysis was repeated to evaluate whether there was any correlation between gp160 concentration, total Env concentration, and gp120:gp160 ratio and neutralization sensitivity to any antibody or plasma sample. This same method was used to evaluate whether there was a correlation between enfuviritide sensitivity of the Q461 variants and the neutralization sensitivity of these variants.

## Results

### Identification of Amino Acid Changes Leading to Neutralization Susceptibility

**Figure 1:**
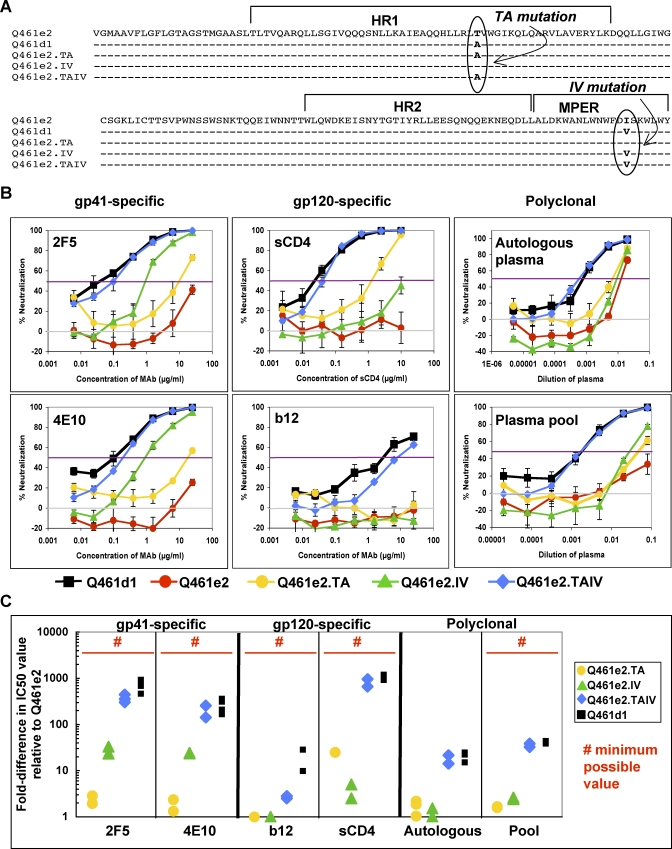
Sequence and Neutralization Profiles of Q461 Viral Variants (A) The predicted amino acid sequences of the portion of gp41 spanning from the fusion peptide to the MPER of the Q461 viral variants are shown. The locations of HR1, HR2, and MPER are indicated. The Q461e2 sequence is shown as a reference, and the locations of the TA and IV mutations within Q461d1 and the Q461e2 mutants are indicated in the oval and marked with an arrow. A dash indicates no change in the amino acid at that position. (B) Neutralization curves of pseudotyped viruses made with the Env variants described in (A). The y-axis represents the percentage of neutralization, the x-axis represents the concentration of MAb or dilution of plasma tested, as appropriate, and the color and symbol key for the different viral pseudotypes is shown at the bottom. The antibody or plasma sample used is indicated in the inset of the graph, and the global specificity above the charts. The purple line indicates 50% neutralization. (C) The fold-difference in the IC50 values for the Q461e2 mutants and the original neutralization-sensitive Q461d1 variant relative to Q461e2 are shown on log scale, and calculated as described in the Methods. Each point represents the value from a single experiment using an independent viral preparation, for two to four replicate experiments for each virus/antibody combination. Each individual experiment was performed in triplicate. When an IC50 value was not reached for the Q461e2 variant because of its neutralization resistance, the fold-difference for that antibody or plasma sample was calculated using the maximum concentration tested as the IC50 value for Q461e2, and thus reveals the minimum possible difference. A red # sign is marked in the graph above these minimum possible values. The color-coding of the different viruses is shown on the right and is the same as in (B). The specificity of the different antibodies is indicated above the graphs, with different specificities separated by bold lines.

Using samples collected as part of a longitudinal cohort study of HIV-1-negative women in Kenya who are at high risk of HIV-1 infection, we previously identified a HIV-1 subtype A-infected participant, Q461, who had a relatively homogenous virus population at 28 d postinfection [20]. One viral variant from that individual, Q461d1, was unusually sensitive to neutralization by the MAbs 2F5, 4E10, and b12 [17]. This variant was also sensitive to sCD4 and to autologous and heterologous plasma samples [17]. Another variant from the same individual, Q461e2, was highly neutralization resistant [17]. There were only four amino acid differences in gp160 between the two variants (Figure S1). In order to determine which of these changes were responsible for the neutralization-sensitive phenotype, we introduced each of these substitutions into the neutralization-resistant Q461e2 Env sequence, and then generated pseudoviruses. Two of the amino acid changes, an asparagine (N) to serine (S) substitution in the V2 region of gp120, and a valine (V) to isoleucine (I) substitution within the membrane spanning domain of gp41, had no effect on the neutralization profile of the resulting virions (unpublished data). However, two other mutations, both within gp41, dramatically altered the neutralization profile of the mutant pseudoviruses. The first mutation, T569A, involved the substitution of a conserved threonine (T) to an alanine (A) within heptad repeat 1 (HR1) of gp41; this mutation is referred to subsequently as TA (Figure 1A). The second mutation was the substitution of a conserved I with a V in the MPER (I675V, referred to as IV), and within the binding site for 4E10, which binds to the NWFDI(T/S) core sequence [29,30].

The TA mutation, when introduced into Q461e2 (Q461e2.TA, gold symbols in Figure 1) resulted in subtle enhancement of neutralization by 2F5 and 4E10 (Figure 1; Table 1). The MAb b12 did not neutralize the Q461e2.TA variant. However, the Q461e2.TA variant was >25-fold more susceptible to sCD4 (Figure 1; Table 1), which inhibits viral entry through interactions with the conformational CD4 binding site on gp120 [31,32]. The Q461e2.TA variant was also slightly more susceptible than Q461e2 to autologous plasma and to a collection of neutralizing antibodies generated by pooling plasma from 30 HIV-1–infected individuals in Kenya (Figure 1; Table 1). Thus, this modified variant was slightly more susceptible than the Q461e2 variant to the mix of antibodies generated by unrelated HIV-1-infected individuals.

**Table 1:**
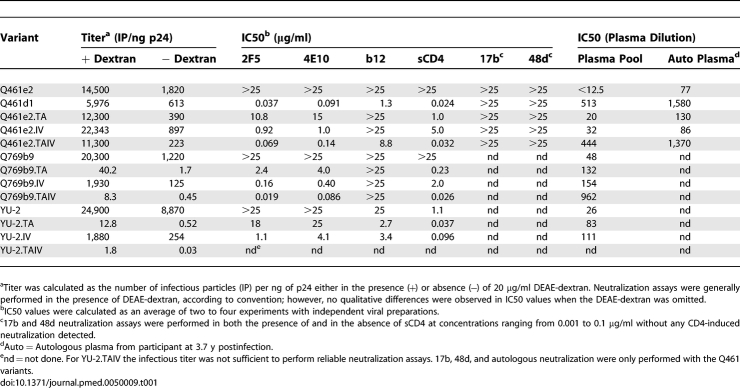
Infectivity and IC50 Values of Viral Variants

The IV mutation (Q461e2.IV, green symbols in Figure 1) also affected the neutralization profiles (Figure 1; Table 1). The Q461e2.IV variant was >27-fold more susceptible to 2F5 and >24-fold more susceptible to 4E10 than the original Q461e2 variant. Q461e2.IV was also >5-fold more susceptible to sCD4, but was not neutralized by b12. Q461e2.IV was modestly more susceptible to autologous plasma and the plasma pool than Q461e2.

The phenotype of the variant with both gp41 changes was markedly more pronounced (Figure 1). Q461e2.TAIV (blue symbols in Figure 1) was >360-fold more susceptible to 2F5, >180-fold more susceptible to 4E10, >780-fold more susceptible to sCD4, and >2.8-fold more susceptible to b12 (Figure 1; Table 1). The TAIV mutation also resulted in 18-fold enhanced susceptibility to autologous plasma and >35-fold enhanced susceptibility to the plasma pool. Thus, these two mutations were synergistic and together led to remarkable neutralization susceptibility to a diverse spectrum of neutralizing antibodies.

### Contribution of Envelope Concentration and Dissociation to Neutralization Susceptibility

We next investigated possible mechanisms that could contribute to the observed enhanced neutralization by antibodies specific for multiple epitopes on both gp41 and gp120. We first evaluated the infectivity of the viral variants. The neutralization-sensitive Q461d1 variant had an approximately 3-fold decrease in infectivity when compared to the neutralization-resistant Q461e2 variant, both in the presence and in the absence of DEAE-dextran (Table 1). However, the equally neutralization-sensitive Q461e2.TAIV had only a negligible difference (1.3-fold) in infectivity when compared to the neutralization resistant Q461e2 variant, indicating that decreased infectivity is unlikely to explain the differences in neutralization observed. In fact, the single mutants had similar (Q461e2.TA) or even increased (Q461e2.IV) infectivity when compared to the parental Q461e2 isolate (Table 1). Infectivity was 32-fold, 25-fold, and 51-fold lower in the absence of DEAE-dextran for the Q461e2.TA, Q461e2.IV, and Q461e2.TAIV variants, respectively.

**Figure 2:**
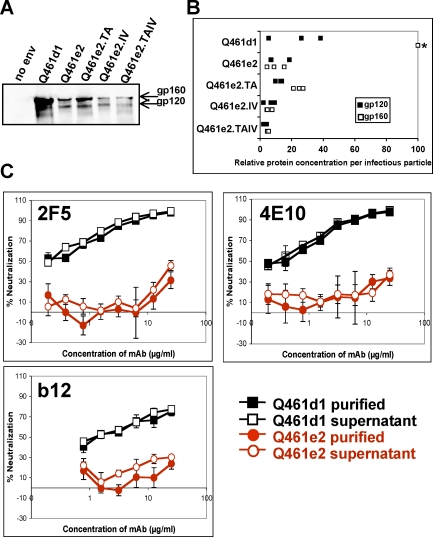
Evaluation of HIV-1 Envelope Concentration and Dissociation of Q461 Envelope Variants (A) A Western blot of purified viral variants is shown. The viral variant tested is indicated along the top; an equal number of infectious particles was loaded for each viral variant. The “No env” negative control was prepared from cell-free viral supernatant produced from Q23Δenv in the absence of a plasmid encoding a viral Env; because this preparation was unable to infect cells, the maximum possible volume was subjected to purification. The gp160 and gp120 bands are marked with an arrow. (B) Relative gp160 and gp120 expression levels calculated from Western blotting are shown for each of three triplicate experiments. The pseudovirus is indicated on the left, gp120 is shown in the black symbols, and gp160 in the open symbols. Levels of protein expression were normalized relative to the gp160 from Q461d1, which was assigned the level of 100, and marked with an asterisk. The Spearman rank test revealed no correlation between either gp120 or gp160 concentration and susceptibility to any MAb or plasma sample (*p* > 0.8 for all comparisons). (C) Neutralization curves of purified (closed symbols) and unpurified viral supernatant (open symbols). Results are shown for both Q461d1 (black squares) and Q461e2 (red circles) with 2F5, 4E10, and b12. As in Figure 1B, the y-axis represents the percentage of neutralization, the x-axis represents the concentration of MAb, and the antibody tested is indicated in the inset.

We then evaluated the concentration of Env protein on the different viral variants (Figure 2A). We normalized the Env content to the number of infectious particles, since neutralization assays were performed with equal numbers of infectious particles. Similar results were obtained when we normalized Env content to p24 content (Figure S2). We observed no relationship by Spearman rank correlation between the concentration of either unprocessed Env, gp160, or gp120 and neutralization sensitivity to any MAb or plasma sample, whether we compared the Env content to the number of infectious particles loaded (Figure 2B) or to the concentration of p24 (Figure S2), with *p* > 0.8 for all comparisons. For example, both the neutralization-sensitive (Q461d1) and neutralization-resistant (Q461e2) variants had similar amounts of gp120 on the pseudoviruses, though they differed dramatically in gp160 concentration. However, the Q461e2.TAIV variant, which is similar to Q461d1 in its neutralization sensitivity, has low concentrations of both gp160 and gp120 (Figure 2A and 2B), indicating that the Env content was not a correlate of neutralization phenotype.

We also considered the possibility that the gp41 mutations altered the interaction between gp120 and gp41, leading to excess Env dissociation from the virion. This excess dissociation could alter the exposure of epitopes on gp41, and could result in antibody binding to the shed gp120 in the viral supernatant. We therefore used a 20% sucrose cushion to purify virus away from shed Env, and evaluated the neutralization sensitivity of purified virions compared to unpurified cell-free supernatants. No differences were observed in the neutralization sensitivity of the purified virus when compared with the cell-free supernatant (Figure 2C), making shedding of gp120 an unlikely cause of the observed differences in neutralization sensitivity. Similarly, we performed neutralization assays with and without DEAE-dextran, and observed no overall differences in neutralization sensitivity its presence or absence (Table 1 and unpublished data). Thus, differences in Env infectivity, Env glycoprotein content on the virions, and Env dissociation rates did not appear to account for differences in the neutralization sensitivity of the viruses.

### Susceptibility of Q461 Variants to HIV-1 Entry Inhibitors

**Figure 3:**
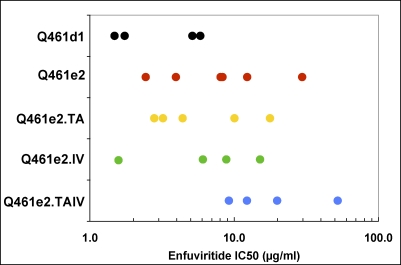
Infectivity and Susceptibility to Enfuviritide of Q461 Envelope Variants IC50 values (in μg/ml) to enfuviritide of the pseudovirus indicated on the left are shown for each of five (Q461e2.TA, Q461e2.IV, Q461e2.TAIV) or six (Q461d1 and Q461e2) replicate experiments; in some cases points overlap. The Spearman rank test revealed no correlation between the mean enfuviritide concentration and mean susceptibility to any MAb or plasma sample (*p* > 0.8 for all comparisons).

Viral Env proteins with enhanced sensitivity to entry inhibitors can also be more susceptible to neutralizing antibodies, likely because sensitivity to entry inhibitors can be a marker of slower fusion, which in turn can lead to prolonged exposure of and access to neutralization epitopes [33–35]. We therefore next examined the sensitivity of these viral variants to enfuviritide, the fusion inhibitor that binds to HR1 and prevents formation of the membrane fusion complex (Figure 3). We did not observe a correlation between enfuviritide sensitivity and neutralization sensitivity by Spearman rank correlation (*p* > 0.8 for all comparisons). Moreover, the two neutralization-sensitive variants, Q461d1 and Q461e2.TAIV, showed the greatest differences in enfuviritide sensitivity, whereas the neutralization-resistant variant Q461e2 was intermediate in its sensitivity. Although the IC50 values to enfuviritide are somewhat high in comparison with subtype B variants found in chronic infection, these are similar to the IC50 values observed among subtype C variants during acute infection [36].

We also evaluated whether the neutralization-sensitive variants were already in an exposed “CD4-bound” conformation by evaluating sensitivity to the CD4-induced antibodies 17b and 48d [37–40]. None of the viral variants were sensitive to these MAbs either in the presence or the absence of sCD4, indicating that a “CD4-bound” conformation did not explain the neutralization sensitivity of the mutant variants (Table 1). Thus, prolonged exposure of neutralization epitopes did not appear to explain neutralization sensitivity.

### Frequency of A and V Substitutions at Positions 569 and 675

An examination of 975 aligned HIV-1 Env sequences at http://www.HIV-1.lanl.gov revealed that T569 is conserved in >93% of sequences and I675 is conserved in >99.5% of sequences. We examined whether the Q461d1 variant, with its mutant T569A and I675V alleles, was selected against in vivo by virtue of its neutralization-sensitive phenotype. Thus, in order to determine the frequency of the TA and IV mutations at baseline, we evaluated 18 full-length Env sequences that were cloned previously by limiting dilution PCR at 28 d postinfection [20]. Only one of 18 variants (6%), the Q461d1 variant, contained both the T569A and I675V mutations. All the other sequences contained the consensus T569 and I675. We next amplified a portion of gp41 encompassing both mutations at 3.7 y postinfection by single-copy PCR, and 24 amplicons were obtained from 84 independent PCRs. All the variants contained the consensus T569 and I675. Thus, the frequency of the TA and IV mutations was <1/24, or <4%, at 3.7 y postinfection.

### Effect of the gp41 TA and IV Mutations in Diverse HIV-1 Envelope Variants

**Figure 4:**
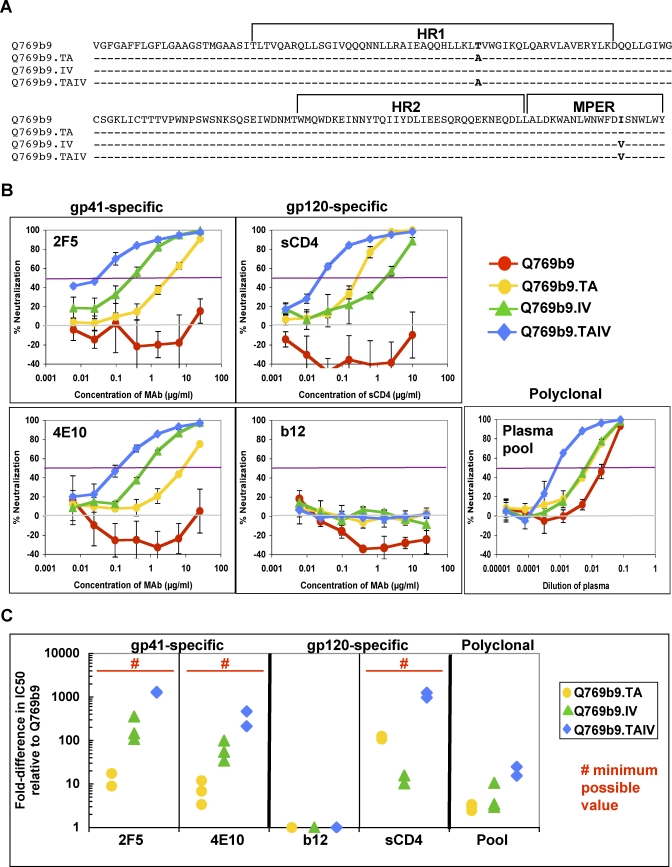
Sequence, Infectious Titer, and Neutralization Profiles of Q769b9 Envelope Variants (A) The predicted gp41 amino acid sequences of Q769b9 and the TA, IV, and TAIV mutants. Layout is as described in the legend to Figure 1A. (B) Neutralization curves of pseudotyped viruses made with the Env variants described in (A) are shown. The layout is as described in the legend to Figure 1B. (C) The -fold difference in the IC50 for the Q769b9 mutants relative to Q769b9 is shown on a log scale as in Figure 1C.

We next evaluated whether introduction of the TA, IV, and TAIV mutations could alter the neutralization profile of unrelated viruses. We selected a subtype A viral variant, Q769b9, that was generally resistant to neutralization by antibodies and plasma samples [17], and introduced these mutations into its sequence (Figure 4A). In this heterologous viral context, the mutations resulted in a decrease in infectivity of the viruses (Table 1). Q769b9.TA demonstrated enhanced susceptibility to 2F5 (>11-fold), 4E10 (>6-fold), sCD4 (>110-fold), and the plasma pool (2.7-fold), though not to b12 (Figure 4; Table 1) when compared with Q769b9. The IV mutation in Q769b9 also resulted in enhanced susceptibility to 2F5 (>160-fold), 4E10 (>60-fold), sCD4 (>12-fold), and the plasma pool (3.2-fold).

The TA and IV mutations together resulted in a remarkable >1,300-fold enhanced susceptibility to 2F5, >290-fold enhanced susceptibility to 4E10, >960-fold enhanced susceptibility to sCD4, and 20-fold enhanced susceptibility to the plasma pool in Q769b9 (Figure 4; Table 1). We did not observe enhanced susceptibility to b12 in the Q769b9.TAIV variant. The magnitude of the changes in neutralization mediated by the two mutations together was similar in the Q461e2 and Q769b9 contexts, although each mutation alone had a more pronounced effect in Q769b9 than in Q461e2 (Figures 1C and 4C). Thus, we found that the gp41 mutations exposed normally shielded neutralization epitopes on this unrelated subtype A virus, although they resulted in a decline in infectivity that was not observed in the Q461 background.

**Figure 5:**
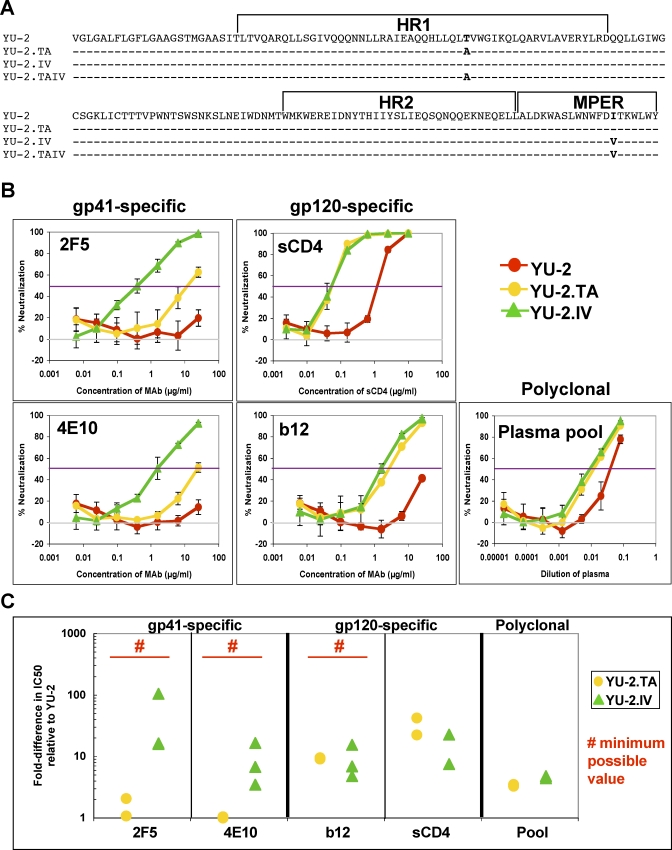
Sequence and Neutralization Profiles of the Subtype B YU-2 Envelope Variants (A) The predicted gp41 amino acid sequences of YU-2 and the TA, IV, and TAIV mutants. Layout is as described in the legend to Figure 1A. (B) Neutralization curves of pseudotyped viruses made with the Env variants described in (A) are shown. The layout is as described in the legend to Figure 1B. (C) The fold-difference in the IC50 for the YU-2 mutants relative to YU-2 is shown on a log scale as in Figure 1C.

Finally, we evaluated whether these changes would also alter neutralization of a subtype B virus by introducing the same amino acid substitutions into a neutralization-resistant subtype B virus, YU-2 (Figure 5A) [41]. Unfortunately, in the YU-2 background, the TAIV mutant decreased the infectivity of the virus below levels that could be used in neutralization assays, and the single mutations also led to a decrease in infectivity (Table 1). The YU-2.TA variant was somewhat more susceptible to 2F5 and 4E10, and much more susceptible to sCD4 (30-fold), b12 (9.3-fold), and the plasma pool (3.2-fold) than was YU-2 (Figure 5B and 5C). The YU-2.IV variant was also more susceptible to 2F5 (>23-fold), 4E10 (>6.1-fold), b12 (7.3 fold), sCD4 (11-fold), and the plasma pool (4.3-fold). The effects of the TA and IV mutations in the YU-2 context were similar in magnitude to those in the Q461e2 context for 2F5 and 4E10 (Figures 1C and 5C). However, the effects of these mutations on susceptibility to b12, sCD4, and the plasma pool was up to 10-fold higher in the YU-2 context than that observed in the Q461e2 context. Thus, the gp41 mutations dramatically enhanced exposure of neutralization epitopes on gp41 and gp120 even individually in the context of an Env protein from a different viral subtype.

## Discussion

Within a longitudinal cohort study we have identified a variant of HIV-1 that naturally presents critical conserved regions of the Env protein to the immune system, permitting the generation of neutralizing antibodies. Here we have identified two amino acid mutations in the gp41 sequence of this variant that conferred this neutralization sensitivity to a broad spectrum of neutralizing antibodies. Transferring the mutations found in this early variant to unrelated variants of HIV-1 resulted in up to 1,000-fold differences in neutralization sensitivities to a wide range of antibodies, indicating that normally shielded neutralization epitopes were now accessible to antibody. Thus, we have identified two mutations that expose the conserved HIV-1 Env regions that are desired targets of protective antibodies to HIV-1. While other studies have shown that gp41 changes can alter neutralization sensitivity [30,42–49], the changes in neutralization susceptibility mediated by the T569A and I675V mutations are unparalleled in their magnitude, breadth, and ability to confer their effects on diverse HIV-1 Env proteins.

The participant from whom we isolated this variant had plasma that was more potently and broadly neutralizing than the plasma pool or any other individual plasma tested [17]. Although we do not know which of her viral variants stimulated the generation of her broad neutralizing antibody response, it is tempting to speculate that the neutralization-sensitive variant exposed conserved epitopes that elicited this response. Because the variant encoding the 675A and 569V mutations was rare at the time of infection in which it was first detected, it is difficult to say whether it ever was a dominant virus in the population. It is not even possible to exclude the possibility that PCR error contributed to these mutations, because only one sequence encoded these mutations. Nonetheless, variants containing the TA and IV mutations appear to present epitopes in a manner that reflects the natural Env structure, because heterologous plasma samples potently neutralize these variants. Given that the Q461d1 variant was highly neutralization-sensitive, we predicted that it would be negatively selected in vivo by the host neutralizing antibody response. Consistent with this prediction, we did not detect this variant at a later time point, 3.7 y postinfection. However, because we detected this variant in only 6% of cloned sequences from this individual at the earlier time point, we cannot distinguish between disappearance of this variant due to stochastic rather than selective events or selection against this variant in vivo by the host antibody response.

These data provide some suggestions as to how the TA and IV mutations found within the Q461d1 variant lead to enhanced neutralization sensitivity. These mutations appear to lead to the exposure of epitopes that are present but normally shielded from the immune system. For instance, although the MPER-specific MAbs 2F5 and 4E10 are broadly neutralizing, some viral variants containing the canonical epitopes, including Q461e2, Q769b9, and YU-2, are resistant to these MAbs, indicating that the epitope is probably shielded [16,17,27,50]. The TA and IV mutations converted these neutralization-resistant variants into neutralization-sensitive variants. This conversion did not occur through changes within the epitope, because the 2F5 epitope was unaltered. Thus, the enhanced sensitivity likely reflects changes in MPER accessibility that allowed both 2F5 and 4E10 access to the viral epitopes that were present. In addition, despite identical gp120 amino acid sequences, viral variants containing the gp41 TA and IV mutations were more susceptible to b12 and sCD4, indicating enhanced exposure of the determinants overlapping the CD4 binding site. The gp41 TA and IV mutations also enhanced exposure of the unidentified neutralization epitopes to which unrelated HIV-1-infected individuals made antibodies. Thus, these two mutations dramatically enhanced exposure of discontinuous conserved neutralization determinants throughout the Env protein. These epitopes included the MPER region and the CD4 binding site, which are desired targets of protective antibodies because of their potential for broad specificity. Understanding how to expose these epitopes through mechanisms such as these gp41 mutations is important to our understanding of how to generate broadly neutralizing antibodies.

The precise mechanism by which these relatively conserved mutations in gp41 exposed neutralization epitopes remains unclear. The levels of infectivity, Env glycoprotein concentration, or Env dissociation cannot fully explain the neutralization susceptibility of the mutant Q461 viruses. In the SIV (simian immunodeficiency virus) system, increasing amounts of Env increased infectivity and decreased sensitivity to antibody-mediated neutralization [51], indicating that decreasing amounts of Env might make a virus easier to neutralize. However, this did not appear to be the mechanism by which the TA and IV mutations enhanced neutralization sensitivity among the Q461 variants, as there were only subtle differences in infectivity and no consistent changes in Env content between the variants that could explain the enhanced neutralization sensitivity. Furthermore, these variants were not neutralized by the CD4-induced antibodies 17b or 48d, suggesting that these mutations did not act by exposing the coreceptor binding site. Despite the location of the TA mutation within HR1, the gp41 mutations did not alter susceptibility to enfuviritide, which is a surrogate marker for differences in fusion kinetics [33]. Finally, the Q461d1 and Q461e2 variants were similarly susceptible to the CCR5 inhibitors PSC-RANTES and TAK-779, indicating that both variants likely had similar affinity for the coreceptor [17]. The absence of changes between variants in their susceptibility to entry inhibitors fails to support the idea that the neutralization-sensitive mutant variants had slower fusion kinetics leading to prolonged exposure of fusion intermediates, because such changes would also result in enhanced sensitivity to enfuviritide, CCR5 inhibitors, and possibly the CD4-induced antibodies 17b and 48d. Instead, since discontinuous epitopes in both gp41 and gp120 are affected by these gp41 mutations, we suggest that these mutations resulted in conformational changes in the Env glycoprotein that exposed shielded neutralization epitopes. Structural studies are needed to evaluate this hypothesis; however, the enhanced exposure of both linear and conformational determinants at sites distant from the mutations argues for a conformational change mediated by these mutations.

The location of the T569A mutation, in particular, lends itself to speculation about how this mutation could lead to exposure of neutralization epitopes. This residue falls within the hydrophobic pocket of HR1, a region which is critical for interaction with HR2 and formation of the fusion complex [52,53]. Although we could not find other examples of substitutions at T569, substitutions in the neighboring regions of gp41 frequently abrogate infectivity, reportedly by disrupting the packing of this hydrophobic pocket and thus interfering with fusion [54,55]**,** and other mutations in this region can modify sensitivity to sCD4 [56]. The TA mutation could therefore alter the packing of this pocket, affecting the overall Env trimeric structure. There is less information on mutations at position 675V. Zwick et al. [30] describe an I675A mutation in which sensitivity to 2F5 and 4E10 was enhanced, and Back et al. [44] describe an I675M/N668S mutation in which sensitivity to sCD4 and anti-V3 antibodies was enhanced, but sensitivity to plasma samples was decreased. Thus, there is some precedent for mutations in both HR1 and the MPER to have an effect on the overall Env trimeric structure; however, this is the first study to suggest that a combination of mutations in these regions can act in synergy to expose multiple antibody epitopes in the context of different viral variants.

The major limitation of this study is the fact that we have not yet elucidated the mechanism by which the neutralization epitopes are exposed in the presence of 675V and 569A. While we were able to investigate the concentration of Env and whether Env was shed, detailed biochemical and structural studies of the trimeric form of these different Env proteins are needed to fully understand the structural changes that contribute to exposure of these key epitopes. Another limitation of this study is that it was performed entirely with pseudoviruses and a single-cycle infection assay using a cell line, rather than replicating virus and primary cells, which are more relevant. While pseudoviruses provide reproducible data, the Env content can differ in pseudoviral preparations compared to PBMC-derived viruses, which can affect neutralization sensitivity [51]. However, in general, we (unpublished data) and others [50] have found that pseudoviruses and PBMC-derived viruses with the same Env protein give qualitatively similar neutralization. Finally, the fact that these variants have not yet been tested as immunogens represents another limitation of this study, as it remains to be determined whether their enhanced exposure of neutralization epitopes will result in enhanced immunogenicity.

This work opens several exciting future directions. Studies of the structure of variants containing these mutations, while technically difficult, may provide significant insights into how these mutations alter the Env protein to present conserved epitopes more effectively. Furthermore, Env-based immunogens containing these gp41 mutations could potentially act as scaffolds to present conserved HIV-1 epitopes such as the CD4 receptor binding site and the MPER region. Previous efforts to enhance targeting of conserved epitopes by using immunogens in which potential glycosylation sites were altered or variable loops removed were disappointing [6,57–59], and other methods to present stabilized conserved regions in immunogens are being tested [11,57,58]. The mutations described here are unique in that they have much more dramatic and universal effects on neutralization sensitivity, and thus epitope exposure, than the other modifications so far tested. In addition, these mutations were identified in the context of a naturally occurring, functional Env variant, and may therefore more accurately present the conserved functional sites in a context relevant to transmitted HIV-1 variants. Thus, incorporation of the gp41 TA and IV mutations into Env-based immunogens may lead to the generation of more broadly neutralizing antibodies directed against conserved regions of HIV-1 Env.

## Supporting Information

Figure S1 Amino Acid Alignment of Q461e2 and Q461d1 HIV-1 Envelope Proteins Predicted amino acid sequence for the neutralization-resistant variant Q461e2 is shown, with a dash in the Q461d1 sequence indicating that the amino acid at that position is conserved. Major functional structures are indicated including the signal peptide, the start of the mature gp120, the five variable regions (V1–V5), the cleavage site between gp120 and gp41, HR1, HR2, MPER, and membrane spanning domain (msd). An oval is placed around the four amino acids that differ between the variants, and the TA and IV mutations are marked with arrows. The TA substitution is at position 569 and the IV mutation is at position 675 according to the HxB2 numbering system.
(45 KB PDF)

Figure S2 Evaluation of Envelope Concentration in Comparison to p24 Levels The Western blots of purified viral variants as shown in Figure 2 was used to determine the relative gp160 and gp120 expression levels compared to p24 levels. The pseudovirus is indicated on the left, gp120 is shown in the black symbols, and gp160 in the open symbols, with error bars representing standard deviation of three independent experiments. Levels of protein expression were normalized relative to the gp160 from Q461d1, which was assigned the level of 100, and indicated by an asterisk.
(22 KB PDF)

Table S1 Primers and PCR Conditions Used in the Generation of HIV-1 Envelope Mutants (21 KB DOC)

### Accession Numbers

No novel genes or proteins were used in this study. The GenBank (http://www.ncbi.nlm.nih.gov/Genbank) accession numbers for Q461d1, Q461e2, and Q769b9 are AF407155, AF407156, and AF407157, respectively [20]. The GenBank accession number for YU-2 is M93258 [41,60].

## Abbreviations

IC50: median inhibitory concentration
MAb: monoclonal antibody
MPER: membrane-proximal external region
PBMC: peripheral blood mononuclear cell
sCD4: soluble CD4
