# Supplementary material for: Enhancing Exposure of HIV-1 Neutralization Epitopes through Mutations in gp41
Source: PLoS Med. 2008 Jan 3;5(1):e9. doi: 10.1371/journal.pmed.0050009 (PMC2174964; doi:10.1371/journal.pmed.0050009)
Supplement: Supplementary file 1 [file pmed.0050009.sg001.pdf]

```
                    Signal peptide <|> gp120
Q461e2    MRVMGIQRNYQHLWRWGTMLLGMLMTCSVTGQWVTVYYGVPVWKDAETTLFCASDAKAYETEK
Q461d1    ---------------------------------------------------------------

Q461e2    HNVWATHACVPTDPNPQEIRLENVTEDFNMWKNSMVEQMNEDIISLWDQSLKPCVKLTPLCVT
Q461e1    ---------------------------------------------------------------

                    V1                                 V2
Q461e2    LNCTDWTNNATSTNQTTPATSEETGVKNCSFNITTELRDKKQKVYSLFYKLDVVQISENNSSN
Q461d1    ----------------------------------------------------------S----

            V2
Q461e2    SSNFTQYRLINCNTSAITQACPKVSFEPIPIHYCAPAGFAILKCNDSVFNGTGPCKNVSTVQC
Q461d1    ---------------------------------------------------------------

                                                              V3
Q461e2    THGIKPVVSTQLLLNGSLAERKVMIRSENITNNAKNIIVQFTKPVNITCIRPGNNTRKSVRIG
Q461d1    ---------------------------------------------------------------

                 V3
Q461e2    PGQAFYATGDITGDIRNAHCVVNRTEWNNTLQKVVEQLREYFPNKTIIFTNSSGGDIEITTHS
Q461d1    ---------------------------------------------------------------

                              V4
Q461e2    FNCGGEFFYCNTSKLFNSRWENNGTANMLKNDTGSNETTLILRCRIKQIINMWQRVGQAMYAP
Q461d1    ---------------------------------------------------------------

                                  V5
Q461e2    PIQGVINCTSNITGLILTRDGGGENDTETFRPGGGDMRDNWRSELYKYKVVKLEPLGVAPTMA
Q461d1    ---------------------------------------------------------------

             gp120 <|> gp41                                HR1
Q461e2    KRRVVEREKRAVGMAAVFLGFLGTAGSTMGAASLTLTVQARQLLSGIVQQQSNLLKAIEAQQH
Q461d1    ---------------------------------------------------------------

           TA mutation
                           HR1
Q461e2    LLRLTVWGIKQLQARVLAVERYLKDQQLLGIWGCSGKLICTTSVPWNSSWSNKTQQEIWNNTT
Q461d1    ----A----------------------------------------------------------

                                                         IV mutation
                      HR2                          MPER          MSD
Q461e2    WLQWDKEISNYTGTIYRLLEESQNQQEKNEQDLLALDKWANLWNWFDISKWLWYIKIFIMVVG
Q461d1    -----------------------------------------------V------------I--

               MSD
Q461e2    GLIGLRIVFAIISVVNRVRQGYSPLSFQIPTPNPEGLDRPGRIEEGGGEQDRTRSIRLVSGFL
Q461d1    ---------------------------------------------------------------

Q461e2    ALAWDDLRSLCLFSYHRLRDFILIVARTVELLGHSSLKGLRLGWEGLKYLGNLLSYWGQELKN
Q461d1    ---------------------------------------------------------------

Q461e2    SATNLLDTTAIAVAGWTDRAIEIVQRIVRAILHIPRRIRQGFERALL
Q461d1    -----------------------------------------------
```
