# Supplementary material for: Enhancing Exposure of HIV-1 Neutralization Epitopes through Mutations in gp41
Source: PLoS Med. 2008 Jan 3;5(1):e9. doi: 10.1371/journal.pmed.0050009 (PMC2174964; doi:10.1371/journal.pmed.0050009)
Supplement: Supplementary file 2 [file pmed.0050009.sg002.pdf]

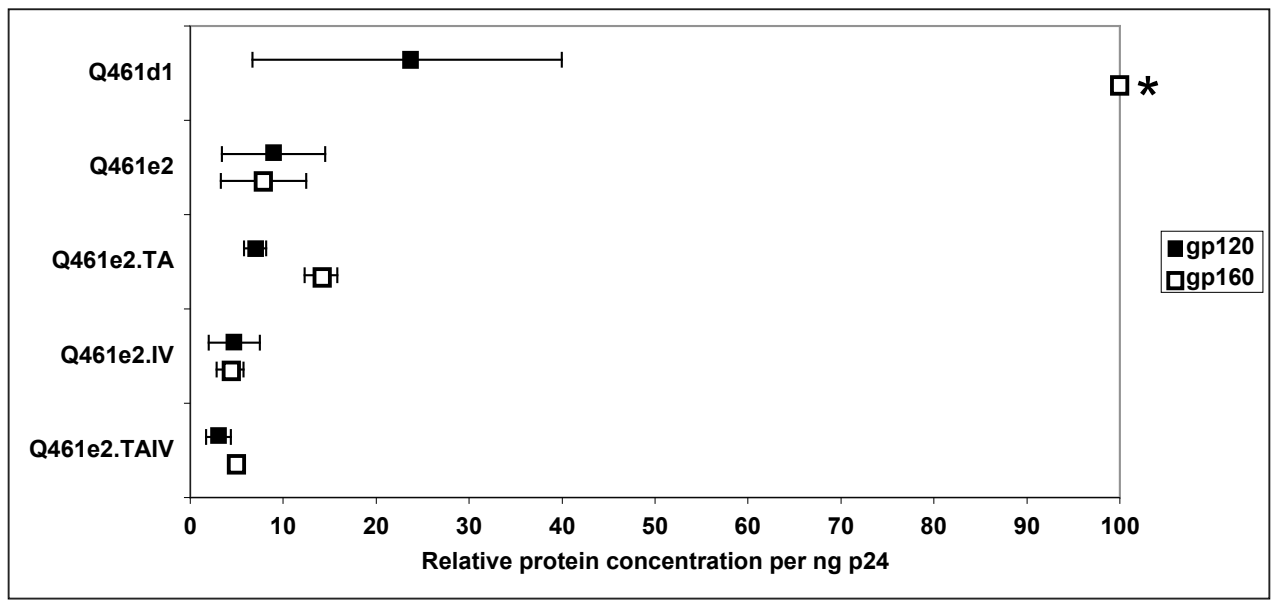
Q461d1
Q461e2
Q461e2.TA
Q461e2.IV
Q461e2.TAIV
*
0
10
20
30
40
50
60
70
80
90
100
Relative protein concentration per ng p24
gp120
gp160
