# Supplementary material for: Enhancing Exposure of HIV-1 Neutralization Epitopes through Mutations in gp41
Source: PLoS Med. 2008 Jan 3;5(1):e9. doi: 10.1371/journal.pmed.0050009 (PMC2174964; doi:10.1371/journal.pmed.0050009)
Supplement: Supplementary file 3 [file pmed.0050009.st001.doc]

Supplemental Table 1. Primers and PCR conditions used in the generation of HIV-1 envelope mutants.

| Mutant | Forward primer (5’-> 3’) | Reverse primer (5’-> 3’) |
| --- | --- | --- |
| Q461TA | GTTGAGACTCGCGGTCTGGGGC | GCCCCAGACCGCGAGTCCCAAC |
| Q461IV | GCAAATCTGTGGAATTGGTTTGACGTATCAAAA TGGCTGTGG | CCACAGCCATTTTGATACGTCAAACCAATTCCA CAGATTTGC |
| Q769TA | GTTGAAACTCGCGGTCTGGGGC | GCCCCAGACCGCGAGTTTCAAC |
| Q769IV | GCAAATCTGTGGAATTGGTTTGACGTATCA | TGATACGTCAAACCAATTCCACAGATTTGC |
| YU-2TA | GTTGCAACTCGCAGTCTGGGGC | GCCCCAGACTGCGAGTTGCAAC |
| YU-2IV | GCAAGTTTGTGGAATTGGTTTGACGTAACA | TGTTACGTCAAACCAATTCCACAAACTTGC |
|  |  |  |
| PCR | 95^o^ x 30 seconds |  |
|  | 95^o^ x 30 seconds, 60^o^ for 1 minute, 68^o^ x 16 minutes | 16 cycles |
